# HMGCS2 Mediation of Ketone Levels Affects Sorafenib Treatment Efficacy in Liver Cancer Cells

**DOI:** 10.3390/molecules27228015

**Published:** 2022-11-18

**Authors:** Fat-Moon Suk, Chien-Ying Wu, Wan-Chun Chiu, Chia-Ying Chien, Tzu-Lang Chen, Yi-Jen Liao

**Affiliations:** 1 Division of Gastroenterology, Department of Internal Medicine, Wan Fang Hospital, Taipei Medical University, Taipei 11696, Taiwan; 2 Department of Internal Medicine, School of Medicine, College of Medicine, Taipei Medical University, Taipei 11031, Taiwan; 3 School of Medical Laboratory Science and Biotechnology, College of Medical Science and Technology, Taipei Medical University, Taipei 11031, Taiwan; 4 School of Nutrition and Health Sciences, College of Nutrition, Taipei Medical University, Taipei 11031, Taiwan; 5 Research Center of Geriatric Nutrition, College of Nutrition, Taipei Medical University, Taipei 11031, Taiwan; 6 Department of Nutrition, Wan Fang Hospital, Taipei Medical University, Taipei 11696, Taiwan; 7 Department of Laboratory Medicine, Wan Fang Hospital, Taipei Medical University, Taipei 11696, Taiwan; 8 Department of Family Medicine, Far Eastern Memorial Hospital, New Taipei City 220, Taiwan

**Keywords:** ketone body, sorafenib, hepatocellular carcinoma

## Abstract

Primary liver cancer is the fifth leading death of cancers in men, and hepatocellular carcinoma (HCC) accounts for approximately 90% of all primary liver cancer cases. Sorafenib is a first-line drug for advanced-stage HCC patients. Sorafenib is a multi-target kinase inhibitor that blocks tumor cell proliferation and angiogenesis. Despite sorafenib treatment extending survival, some patients experience side effects, and sorafenib resistance does occur. 3-Hydroxymethyl glutaryl-CoA synthase 2 (HMGCS2) is the rate-limiting enzyme for ketogenesis, which synthesizes the ketone bodies, β-hydroxybutyrate (β-HB) and acetoacetate (AcAc). β-HB is the most abundant ketone body which is present in a 4:1 ratio compared to AcAc. Recently, ketone body treatment was found to have therapeutic effects against many cancers by causing metabolic alternations and cancer cell apoptosis. Our previous publication showed that HMGCS2 downregulation-mediated ketone body reduction promoted HCC clinicopathological progression through regulating c-Myc/cyclin D1 and caspase-dependent signaling. However, whether HMGCS2-regulated ketone body production alters the sensitivity of human HCC to sorafenib treatment remains unclear. In this study, we showed that HMGCS2 downregulation enhanced the proliferative ability and attenuated the cytotoxic effects of sorafenib by activating expressions of phosphorylated (p)-extracellular signal-regulated kinase (ERK), p-P38, and p-AKT. In contrast, HMGCS2 overexpression decreased cell proliferation and enhanced the cytotoxic effects of sorafenib in HCC cells by inhibiting ERK activation. Furthermore, we showed that knockdown HMGCS2 exhibited the potential migratory ability, as well as decreasing zonula occludens protein (ZO)-1 and increasing c-Myc expression in both sorafenib-treated Huh7 and HepG2 cells. Although HMGCS2 overexpression did not alter the migratory effect, expressions of ZO-1, c-Myc, and N-cadherin decreased in sorafenib-treated HMGCS2-overexpressing HCC cells. Finally, we investigated whether ketone treatment influences sorafenib sensitivity. We showed that β-HB pretreatment decreased cell proliferation and enhanced antiproliferative effect of sorafenib in both Huh7 and HepG2 cells. In conclusion, this study defined the impacts of HMGCS2 expression and ketone body treatment on influencing the sorafenib sensitivity of liver cancer cells.

## 1. Introduction

Liver cancer remains a rapidly increasing malignancy, and it currently ranks as the fifth leading cause of cancer mortality in men [1]. The major type of primary liver cancer is hepatocellular carcinoma (HCC) which is highly associated with viral infections, excess alcohol consumption, metabolic syndromes, nonalcoholic fatty liver disease (NAFLD), and cirrhosis [2]. Chronic hepatitis, the precursor of cirrhosis and HCC, is the result of hepatic wound-healing reactions, which alter the normal function of hepatocytes. Furthermore, patients with chronic cirrhosis are at high risk of developing HCC, which was reported to exhibit poor prognoses [3]. Liver biopsies with ultrasound and computed tomography (CT) are used to detect nodule and tumor tissues. Serum analyses of alanine aminotransferase (ALT), aspartate aminotransferase (AST), and the HCC biomarker, α-fetoprotein (AFP), are commonly used for clinical screening. Surgical resection is a recommended treatment option in patients with early-stage HCC, while targeted drug treatment of liver cancer is used in patients with advanced HCC. Sorafenib, the first-line drug for liver cancer is used for treatment; however, if the treatment is ineffective or sorafenib resistance occurs, a second line drug for HCC will be used. Ultimately, supportive treatment is a better option for terminal HCC patients [4].

Sorafenib is a multi-target kinase inhibitor that blocks tumor cell proliferation and angiogenesis via inhibiting the activity of kinases in the mitogen-activated protein kinase (MAPK) pathway and vascular endothelial growth factor receptors (VEGFRs) [4,5]. Sorafenib is also involved in alternating multiple kinases in epithelial-mesenchymal transition (EMT) pathways and hypoxia-inducible pathways [5]. Previous studies indicated that sorafenib treatment can enhance overall survival in advanced HCC patients by 3~5 months; however, some patients had side effects like palmar-plantar erythrodysesthesia (PPE), diarrhea, hypertension, depraved appetite, and ultimate development of sorafenib resistance [5,6].

Ketone body (KB) metabolism is an alternative energy utilization pathway that is due to an insufficiency of glucose consumption or to prolonged fasting, and it includes ketogenesis and ketolysis. 3-Hydroxy-3-methylglutaryl-CoA synthase 2 (HMGCS2) expression may trigger ketogenesis [7], and subsequently generate the following three KBs in hepatic mitochondria: β-hydroxybutyrate (β-HB), acetoacetate (AcAc), and acetone. The ratio of β-HB to AcAc is nearly 4:1 under normal conditions [8]. Ketolysis is the process of KB reutilization which results in the formation of acetyl-CoA from β-HB and AcAc [9]. Furthermore, KB metabolism plays an important role in connecting several energetic metabolic pathways such as the tricarboxylic acid (TCA) cycle, glycolysis, and de novo lipogenesis [9,10,11,12,13].

KB treatment is generally used in some brain diseases, such as epilepsy and glioblastoma multiforme (GBM). KBs shift the glucose metabolic mechanism, additionally, both β-HB and AcAc were illustrated the positive effects in brain disease. β-HB was associated with the repairment of mitochondrial dysfunction in brain disease [14,15], and AcAc was linked to inhibit the cell viability in GBM cell lines [12]. A previous study demonstrated that the KB level was elevated by sodium-glucose cotransporter-2 (SGLT2) inhibitors which further induced KB utilization in the kidneys and subsequently inhibited hyperactivation of mammalian target of rap amycin complex 1 (mTORC1) that occurs in proteinuric renal disease [16]. Circulating KBs are known to be potential biomarkers of heart failure and the deterioration of arrhythmogenic cardiomyopathy [17,18]. Recent studies focused on the efficacy of KB treatment on other cancers [19,20,21]. Dietary-mediated KBs were illustrated to reprogram metabolic alterations in pancreatic cancer cells, which further reduced tumor growth and cachexia in cell line models [21]. KBs helped enhance radio-chemotherapeutic responses in lung cancer xenografts by accumulating oxidative stress [20]. Application of a ketogenic diet increased the plasma β-HB level which afterward expressed an antiproliferative effect on human gastric cancer cells in nude mice [19]. However, whether HMGCS2-mediated ketone levels alter the sensitivity of human HCC cells to sorafenib treatment remains unclear.

## 2. Results

### 2.1. Knockdown HMGCS2 Enhanced Cell Proliferation and Attenuated the Cytotoxicity Effect of Sorafenib via Disturbing the MAPK and Akt Pathway

**Figure 1:**
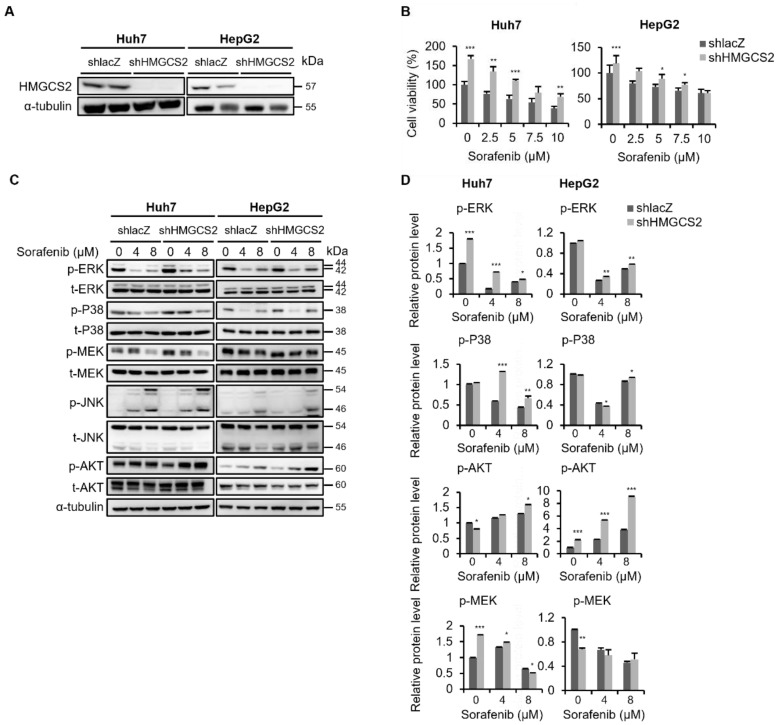
Knockdown of the HMGCS2 gene increased cell proliferation and decreased sorafenib sensitivity through promoting the mitogen-activated protein kinase (MAPK) and Akt signaling pathway in Huh7 and HepG2 cells. (**A**) Western blot analysis of HMGCS2 knockdown in Huh7 and HepG2 cells. (**B**) shlacZ control and HMGCS2-knockdown Huh7 and HepG2 cells were treated with 0, 2.5, 5, 7.5, and 10 μM sorafenib for 48 h, and an MTT assay was conducted to detect cell viability. (**C**) shlacZ control and HMGCS2-knockdown cells were treated with sorafenib (0, 4, and 8 μM) for 48 h. phosphorylated (p)- and total (t)- MAPK- and AKT-related signaling cascades were evaluated by Western blotting. α-Tubulin was used as an internal control. The representative data are obtained from three independent experiments. (**D**) Western blot images from Figure 1C were quantified by using the ImageJ software. * *p* < 0.05; ** *p* < 0.01; *** *p* < 0.001 vs. the shlacZ control. Data are shown as the mean ± SD.

Our previous study established HMGCS2 knockdown and HMGCS2 overexpressing HCC cells and showed that the expression of HMGCS2 and β-HB level affected the progression of HCC [22,23]. In this study, to determine whether HMGCS2 expression affected sorafenib sensitivity in human HCC cells, we treated HMGCS2 knockdown cells (Figure 1A) with sorafenib. Compared to shlacZ control cells, HMGCS2 knockdown cells showed higher proliferative ability in both sorafenib-treated and -untreated Huh7 and HepG2 cells (Figure 1B). A previous study indicated that sorafenib treatment decreased cancer cell proliferation via inhibiting the Ras/RAF/MEK/ERK pathway [5], and in Figure 1C,D we show that the phosphorylation of ERK, P38, and MEK declined with sorafenib treatment in shlacZ control cells. However, this inhibitory effect was attenuated in HMGCS2 knockdown cells. On the other hand, the expression of p-AKT prominently increased in sorafenib-treated HMGCS2 knockdown cells as compared with the control cells (Figure 1C,D).

### 2.2. Overexpression of HMGCS2 Inhibited ERK Activation and Enhanced Sorafenib’s Effects in Huh7 and HepG2 Cells

**Figure 2:**
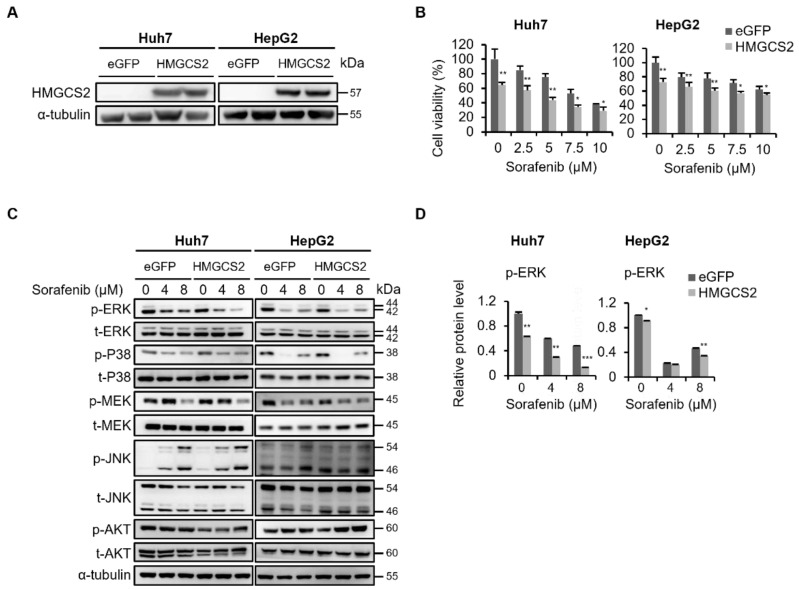
Overexpression of HMGCS2 in sorafenib-treated Huh7 and HepG2 cells exhibited an inhibitory effect on mitogen-activated protein kinase (MAPK)/extracellular signal-regulated kinase (ERK) activation. (**A**) Western blot analysis of HMGCS2 overexpression in Huh7 and HepG2 cells. (**B**) Enhanced green fluorescent protein (eGFP) control and HMGCS2-overexpressing Huh7 and HepG2 cells were treated with 0, 2.5, 5, 7.5, and 10 μM sorafenib for 48 h, and an MTT assay was conducted to detect the cell viability. (**C**) eGFP control and HMGCS2-overexpressing cells were treated with sorafenib (0, 4, and 8 μM) for 48 h. phosphorylated (p)- and total (t)- MAPK- and AKT-related signaling cascades were evaluated by Western blotting. α-Tubulin was used as an internal control. The representative data are obtained from three independent experiments. (**D**) Western blot images from Figure 2C, quantified by using the ImageJ software. * *p* < 0.05; ** *p* < 0.01; *** *p* < 0.001 vs. the eGFP control. Data are shown as the mean ± SD.

To clarify whether HMGCS2 overexpression enhanced sorafenib’s effects on HCC cells, we used HMGCS2 overexpressing Huh7 and HepG2 cells (Figure 2A). Compared to enhanced green fluorescent protein (eGFP) control cells, HMGCS2 overexpression expressed lower proliferative ability in both sorafenib-treated and -untreated Huh7 and HepG2 cells (Figure 2B). Next, we used Western blotting to analyze whether overexpression of the HMGCS2 gene influenced sorafenib sensitivity by transforming expression of the MAPK and Akt pathway. Compared to eGFP control cells, p-ERK and p-MEK expressions had decreased in sorafenib-treated HMGCS2-overexpressing cells (Figure 2C,D). Nonetheless, expressions of p-P38, p-JNK, and p-Akt exhibited no significant discrepancies between sorafenib-treated eGFP and HMGCS2-overexpressing cells. Based on results in Figure 1 and Figure 2, HMGCS2 gene expression affects sorafenib sensitivity due to disturbing the MAPK/Akt pathway in HMGCS2 knockdown cells, whereas inhibition of MAPK/ERK activation was found in HMGCS2-overexpressing cells.

### 2.3. Knockdown HMGCS2 Diminished the Sorafenib-Induced Inhibition of Migration and the Epithelial-Mesenchymal Transition (EMT) Pathway in HCC Cells

**Figure 3:**
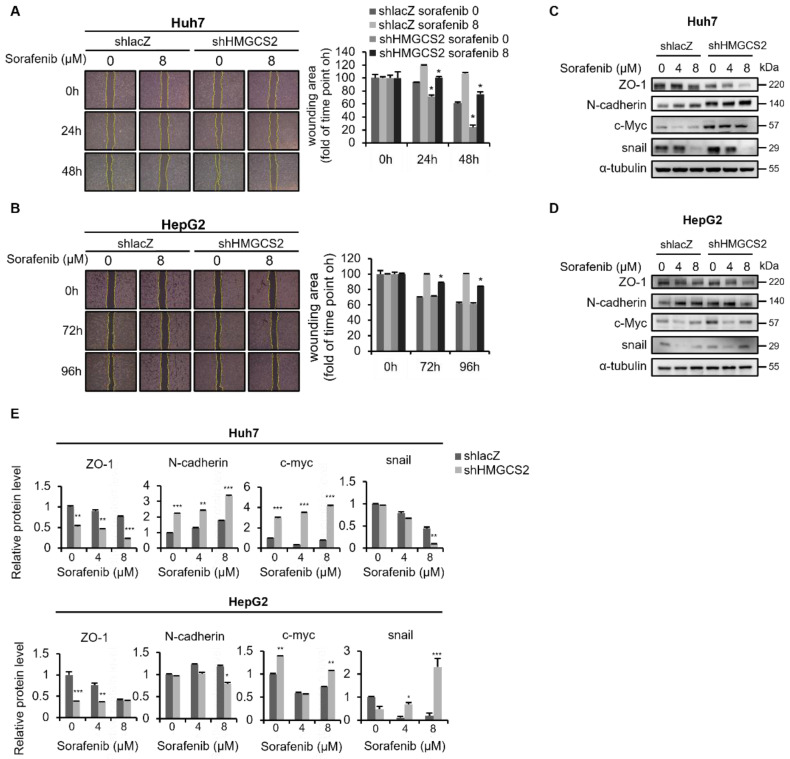
HMGCS2-knockdown enhanced the migratory ability and epithelial-mesenchymal transition of sorafenib-treated Huh7 and HepG2 cells. (**A**,**B**) HMGCS2-knockdown HCC cells (4.3 × 10^5^ cells/well of Huh7 cells and 10^6^ cells/well of HepG2 cells) were seeded in ibidi culture inserts and treated with 0 and 8 μM sorafenib. Images were collected at the indicated time point, and the wound-healing areas were measured with ImageJ software. (**C**,**D**) shlacZ control and shHMGCS2 Huh7 and HepG2 cells were treated with sorafenib at concentration of 0, 4, and 8 μM for 48 h. Protein expression levels of zonula occludens (ZO)-1, N-cadherin, c-Myc, and Snail were evaluated by Western blotting. α-Tubulin was used as a loading control. The representative data are obtained from three independent experiments. (**E**) Western blot images from Figure 3C,D, quantified by ImageJ software. * *p* < 0.05; ** *p* < 0.01; *** *p* < 0.001 vs. the shlacZ control. Data are shown as the mean ± SD.

Sorafenib treatment also inhibited tumor metastasis by inhibiting the EMT pathway [24,25]; however, whether downregulation of the HMGCS2 gene altered sorafenib-induced EMT inhibition remains unknown. The wound-healing migration assay is particularly suitable for studies the effects of cell migration, especially in cancer cells metastasis [26]. A wound-healing migration assay showed that sorafenib treatment inhibited the migration of Huh7 and HepG2 cells; however, this phenomenon was attenuated in HMGCS2 knockdown cells (Figure 3A,B). In contrast to sorafenib-treated shlacZ control cells, shHMGCS2 cells treated with 8 μM sorafenib showed smaller wound areas (Figure 3A,B). We also found that knockdown HMGCS2 decreased expression of the ZO-1 epithelial marker, and increased expressions of the mesenchymal markers, N-cadherin, Snail, and c-Myc (Figure 3C–E), which implied that knockdown HMGCS2 inhibited sorafenib-induced suppression of the EMT pathway.

### 2.4. HMGCS2 Overexpression Had No Effect on the Migration of Sorafenib-Treated Cells

**Figure 4:**
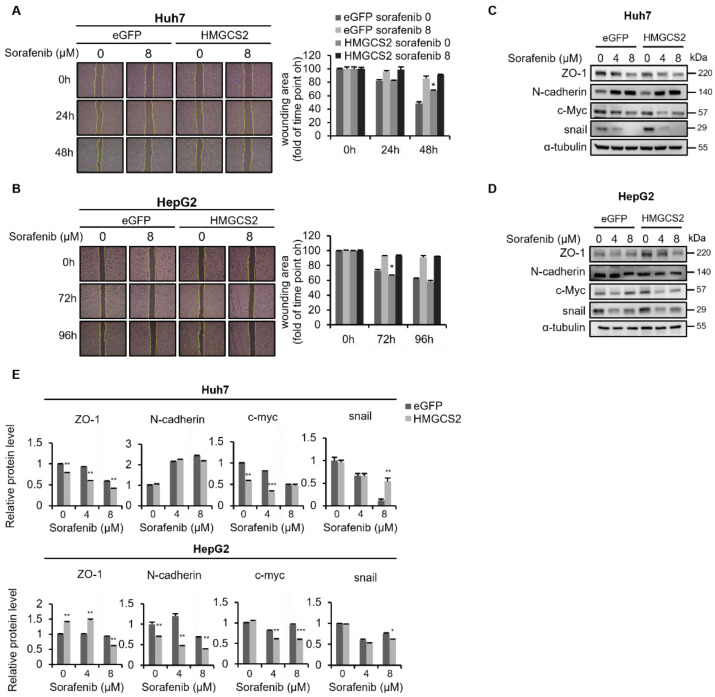
HMGCS2 overexpression did not affect the migratory ability but decreased expressions of N-cadherin and c-Myc in both sorafenib-treated Huh7 and HepG2 cells. (**A**,**B**) HMGCS2-overexpressing cells (4.3 × 10^5^ cells/well of Huh7 cells and 10^6^ cells/well of HepG2 cells) were seeded in ibidi culture inserts and treated with 0 and 8 μM sorafenib. Images were collected at the indicated time points and wound-healing areas were measured with ImageJ software, as shown in the column charts. (**C**,**D**) Cells were treated with sorafenib at concentrations of 0, 4, and 8 μM for 48 h. Protein expression levels of zonula occludens (ZO)-1, N-cadherin, c-Myc, and Snail were evaluated by Western blotting. α-Tubulin was used as a loading control. The representative data are obtained from three independent experiments. (**E**) Western blot images from Figure 4C,D were quantified by ImageJ software. * *p* < 0.05; ** *p* < 0.01; *** *p* < 0.001 vs. the enhanced green fluorescent protein (eGFP) control. Data are shown as the mean ± SD.

Next, we analyzed whether HMGCS2 gene overexpression promoted the sorafenib-induced migratory-inhibitory effect. Without sorafenib treatment, HMGCS2-overexpressing cells demonstrated less migratory ability than eGFP control cells (Figure 4A,B). Although there was no significant difference in migration between eGFP control and HMGCS2-overexpressing cells under sorafenib treatment, protein levels of N-cadherin and c-Myc were lower in HMGCS2-overexpressing cells (Figure 4C–E). Collectively, dysregulation of the HMGCS2 gene altered sorafenib-induced inhibition of tumor migration through regulating expressions of epithelial and mesenchymal markers.

### 2.5. Pretreatment with a Ketone Body (β-HB) Decreased the Cell Proliferative Ability and Enhanced the Cytotoxic Effect of Sorafenib in HCC Cells

**Figure 5:**
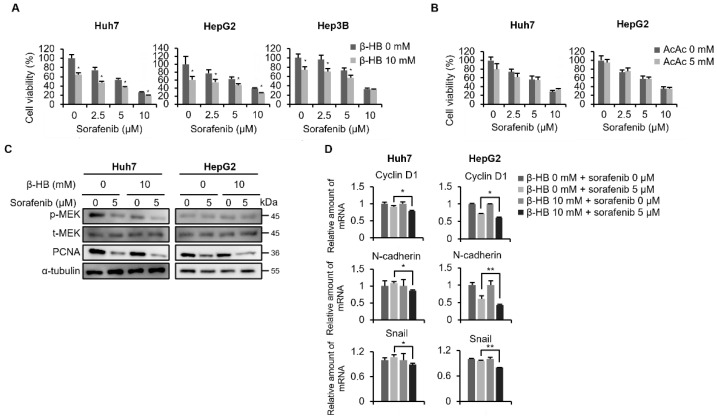
β-Hydroxybutyrate (β-HB) pretreatment decreased cell proliferative ability and enhanced the sorafenib sensitivity in hepatocellular carcinoma (HCC) cells. (**A**,**B**) Huh7, Hep3B, and HepG2 cells were maintained in β-HB- or AcAc-supplemented medium for 2 days, and afterwards cells were seeded in 96-well plates and treated with 0, 2.5, 5, and 10 μM sorafenib for 48 h. An MTT assay was conducted to detect cell viability. (**C**) Huh7 and HepG2 cells were treated with sorafenib (0 or 5 μM) combined with β-HB (0 or 10 mM) for 48 h, phosphorylated (p)-, total (t)-MEK and proliferating cell nuclear antigen (PCNA) were evaluated by Western blotting. α-Tubulin was used as an internal control. (**D**) mRNA expressions of Cyclin D1, N-cadherin, and Snail in sorafenib combined with β-HB treated Huh7 and HepG2 cells were analyzed by QPCR. * *p* < 0.05; ** *p* < 0.01 vs. the β-HB or AcAc free control. Data are shown as the mean ± SD. The representative data are obtained from two independent experiments.

Since HMGCS2 is the rate-limiting enzyme for ketogenesis [7], we investigated whether HMGCS2 expression influenced sorafenib sensitivity under ketone treatment. KBs exist in three forms in the body: β-HB, AcAc, and acetone. β-HB is the most abundant circulating KB at percentages of β-HB to AcAc of around 78%:20%. According to previous study, after prolonged fasting or exercise, the circulating total ketone body concentrations would rise to appropriately 1 mM in healthy adults, and 20 mM was defined as pathological states in diabetic ketoacidosis [11]. Therefore, we chose 10 mM as our working dose. We first maintained HCC cells in medium with/without β-HB or AcAc for 2 days, and then cells were subjected to sorafenib treatment. As shown in Figure 5A, β-HB pretreatment decreased the proliferative ability and enhanced the cytotoxic effects of sorafenib in HCC cells, while there was no distinct difference in an AcAc-pretreated condition (Figure 5B). To investigate the mechanisms behind the increase of sorafenib-induced cytotoxicity in β-HB pretreatment study, we used Western blot and quantitative real-time polymerase chain reaction (QPCR) assay to confirm the influence in proliferative and migratory pathways. As shown in Figure 5C, β-HB pretreatment significantly decreased the expression of proliferating cell nuclear antigen (PCNA) in sorafenib-treated Huh7 and HepG2 cells, whereas the phosphorylation of MEK was only decreased in β-HB pretreated Huh7 cells. The expression of cyclin D1, a downstream of ERK [27], was also decreased in β-HB combined with sorafenib treated HCC cells (Figure 5D). Regarding the mesenchymal markers, we found that β-HB pretreatment diminished the N-cadherin and Snail expression (Figure 5D). These data implied that β-HB treatment may decrease the proliferative ability and enhance the antiproliferative potential of sorafenib in HCC cells.

## 3. Discussion

Regarding whether HMGCS2 expression or β-HB co-treatment affected the cytotoxicity of sorafenib in HCC cells, we tried to use two different normalized viewpoints. We first analyzed the normalized target to 100% with 0 µM sorafenib in MTT cell proliferation assay, but there was no difference between shHMGCS2 vs. shlacZ, HMGCS2-overexpress vs. eGFP, as well as β-HB-treated vs. un-treated cells. However, we found that the cell proliferation was inhibited in β-HB pre-treated HCC cells and enhanced in HMGCS2 knockdown cells. To clarify its underlying effect focus on proliferation between the two groups, we normalized to its shlacZ/eGFP or β-HB-untreated cells. We found that even in sorafenib-treated condition, the cell proliferation was higher in shHMGCS2 cells, whereas lower in β-HB pre-treated cells. These data implying that HMGCS2 downregulation or β-HB pre-treatment may influence HCC cells proliferation and then affect the effectiveness of sorafenib.

MAPK/Akt signaling pathway is associated with the proliferation of cancer cells, specifically in HCC [28,29,30]. To inhibit the proliferation of HCC cells, sorafenib can effectively target the Raf/MEK/ERK pathway [5]. Our data found the attenuation of sorafenib sensitivity in knockdown HMGCS2 HCC cell lines through activating the MAPK/Akt pathway (Figure 1). Nagai et al. reported that the expression of hepatocyte growth factor promotes the progression of liver cancer through activating MAPK pathway, which further expressed transformational change and increased mesenchymal markers in Huh7 and HepG2 cells [31]. In addition, EGF-like repeat and discoidin I-like domain-containing protein 3 (EDIL3), a regulator of EMT, was illustrated to induce the ERK-associated proliferative signal through interactions with αvβ integrin in HCC [32]. Hence, the crosstalk between MAPK and EMT signaling are activating in the hepatocellular carcinogenesis [33]. According to our data, HMGCS2 down-regulation attenuates the antiproliferative and antimigratory effects of sorafenib through deterioration of MAPK/Akt/EMT pathways (Figure 1 and Figure 3).

In this study, we knockdown or overexpress the HMGCS2 gene in both Huh7 and HepG2 cells to study whether HMGCS2 expression affects the effectiveness of sorafenib. Although the antiproliferative effects of sorafenib were attenuated in both HMGCS2-knockdown Huh7 and HepG2 cells, some molecules changed pattern were different between Huh7 and HepG2 cells. These disparities may be explained by the characteristics and genetic variants in Huh7 and HepG2 cells. HepG2 expresses wild type p53, whereas Huh7 harbors mutated p53, which can trigger differential effectiveness of sorafenib related signals in liver cancer cells [34]. Furthermore, the discrepancy of drug-metabolizing genes expression between Huh7 and HepG2 may also affect drug transport related signals [35,36]. We also detected p-P38 significantly decreased in 4 µM sorafenib treatment, but re-increased in 8 µM sorafenib in HepG2 cells. This specific phenomenon is only observed in HepG2 cells. Although we cannot explain the underlying mechanism, this disparity may be due to cell-specific effects or other compensatory effects in HepG2 cells. Further studies are needed to investigate the regulation of P38 at low and high doses of sorafenib.

Recently, the potential of ketone treatment in cancer are rapidly emerging. Although the dose selection and its ketoacidosis may be a concern issue in the clinical, the advantages of ketone salt or ketone ester supplementation in diets have been reported in several animal and clinical studies [37]. Accordingly, the nutritional supplement ketone salt or ketone ester may be safe, effective, and commercially available. Since the chemical structure of our β-HB used in this study is a sodium salt-conjugate β-HB, we believe that this compound may be suitable for treatment in HCC cells. In the beginning of our study, we examined the cytotoxic effects of different dosages of β-HB in HCC cells. We found that 2.5 and 5 mM β-HB did not induce a cytotoxic effect in HCC cells (Supplementary Figure S1); thus, 10 mM was used in our study. Other studies also demonstrated the therapeutic effect of 10 mM β-HB in treating human HCC cell lines and inhibiting the activation of NLRP3 inflammasome in bone marrow-derived macrophages [38,39].

**Figure 6:**
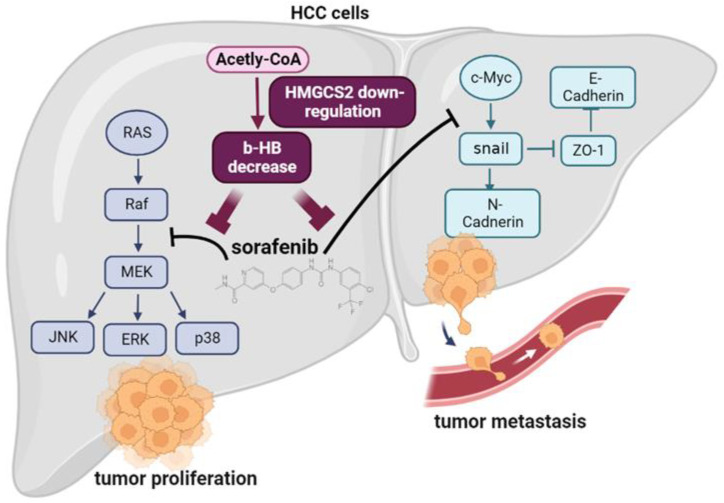
Proposed model demonstrating the mechanisms of HMGCS2 downregulation mediating ketone reduction in influencing the sorafenib treatment efficacy of hepatocellular carcinoma (HCC) cells. The image was created with BioRender.

HMGCS2 was found to be expressed in normal liver, skeletal muscle, heart, pancreas, testis, and colon tissues [40]. In human cancers, HMGCS2 expression differs. Higher HMGCS2 expression was found in estrogen receptor-negative breast cancer [41] and aggressive prostate cancer [42]. In poorly differentiated colon cancer, HMGCS2 protein expression was downregulated [43], while higher HMGCS2 expression caused poor susceptibility of rectal cancer to chemoradiotherapy [44]. In liver cancer, HMGCS2 expression was lower in HCC tissues [45]. Our recent publication showed that HMGCS2-mediated ketone production influenced HCC clinicopathological progression through regulating c-Myc/cyclin D1 and caspase-dependent signaling [22]. We further proved that HMGCS2 downregulation attenuates the protective effect of a ketogenic diet by shifting ketone production to enhance de novo lipogenesis in HCC [23]. However, whether HMGCS2-mediated ketone production governs sorafenib sensitivity in HCC is still unclear. In this study, we showed that HMGCS2 downregulation decreased the antiproliferative and anti-migratory effects of sorafenib in HCC cells (Figure 6), while additional ketone (β-HB) supplementation enhanced the cytotoxicity of sorafenib toward HCC cells. Previously, β-HB was reported to increase cisplatin-induced apoptosis in HepG2 cells [38]. These data support β-HB possibly acting as a new adjuvant agent for HCC chemotherapy. Connections between ketone treatment and cancer are recently rapidly emerging [37]. β-HB is reported to express antiproliferative effect through activating β-HB-Har2-Hopx axis in colorectal cancer [46,47]. Recent study had pointed out the positive effect of KB treatment in glioblastomas [48]. Stimulating the production of β-HB in melanoma and glioblastoma cells by a peroxisome proliferator-activated receptor-α (PPARα) agonist can cause tumor cell growth arrest [49]. Moreover, β-HB can be induced by PPARα activation in the liver partially contributed to the antitumor effect of apatinib [50]. In pancreatic cancer cells, treatment with KBs can inhibit tumor cell growth, proliferation, and glycolysis [21]. Taken together, these data also support that ketone treatment not only can be used in liver cancer, but also can be used to treat other cancers.

A ketogenic diet, the main resource for supplying KBs in vivo, was previously illustrated to play a beneficial role in heart disease since the KBs sustain the proliferation of cardiac endothelial cells [51]. Under the condition of prolonged fasting or insufficient carbohydrates, alternative energetic supplementation is triggered in the liver, and one of the major metabolic pathways is ketogenesis, which catabolizes fatty acids and further elevates levels of KBs in plasma and the brain. Otherwise, KBs can also be supplied through the consumption of a ketogenic diet or ketone administration [14]. Our laboratory previously indicated that a high fat, low-carbohydrate ketogenic diet caused the deterioration of CCl_4_- and thioacetamide (TAA)-induced liver fibrosis in mice through accumulating quantities of cholesterol in the liver [52]; moreover, it was also reported to be associated with atherosclerotic cardiovascular disease since a ketogenic diet helped increase levels of low-density lipoprotein cholesterol [53]. Due to certain side effects of ketogenic diets, more and more research has focused on directly administrating KBs, which not only increased KB levels within 5 h which was the same as 2-week consumption of a ketogenic diet [54,55], but also confirmed that the mechanisms was only influenced by KBs rather than other metabolic features induced by ketogenic diets [56]. Ketone esters, which are metabolized to the KBs, β-HB and AcAc, alleviated tumor cell viability and prolonged survival of VM-M3 mice with systemic metastatic cancer [57]. β-HB and AcAc are the two major KBs circulating in vivo, found in an approximately 4:1 ratio, and previous studies indicated that β-HB rather than AcAc exhibited antitumor effects in various cancers [38,58,59,60]. Our data revealed the antiproliferative and antimigratory effects of HMGCS2 overexpression, which could be linked to the presence of β-HB-induced cytotoxic effects on human HCC cells. Collectively, HMGCS2 expression and the β-HB quantity validated the positive potential for treating HCC.

## 4. Materials and Methods

### 4.1. Cell Culture and Viral Infection

The human (HepG2 and Huh-7) HCC cell lines were cultured in Dulbecco’s modified Eagle medium (DMEM; Gibco, Grand Island, NY, USA) replenished with 10% heat-inactivated fetal bovine serum (FBS; Hyclone, Logan, UT, USA), streptomycin (100 μg/mL), penicillin (100 U/mL), nonessential amino acids (0.1 mM), and L-glutamine (2 mM) at 37 °C in a 5% CO_2_ incubator. Huh-7 belongs to well differentiated HCC cell line, while HepG2 belongs to the hepatoblastoma line. Genetically, HepG2 presents wild type p53 gene, and usually serves as the standard of drug transport study, while Huh-7 possesses the mutant p53 and frequently uses as the alternative to HepG2 in drug metabolism [35]. Establishment of the cell lines with stable HMGCS2 overexpression and knockdown were complied with our previous publications [22,23]. The origin of LacZ was from E. coli β-galactosidase, and shlacZ did not target any human or mouse genes; the enhanced green fluorescent protein (eGFP) was used as the overexpressing control strategy in this study. The origin of these plasmids was obtained from the National RNAi Core Facility (Academia Sinica, Taipei, Taiwan). HMGCS2 knockdown, HMGCS2-overexpressing and their controlled (shlacZ and eGFP) cells were cultured in DMEM supplemented with 10% FBS and 1 μg/mL puromycin. Western blotting was used to verify the HMGCS2 expression in HMGCS2 knockdown or overexpressing HCC cells.

### 4.2. Cell Viability Assay

Human HCC cells (1500 cells/well) were seeded in 96-well plates and switched to fresh medium with different concentrations of sorafenib (ApexBio, Houston, TX, USA) or β-HB (H6501, Sigma-Aldrich, St. Louis, MO, USA). The treatment dosage of sorafenib was referring to other references which indicated that 10 μM of sorafenib is the clinical achievable concentration [61,62]. After incubation for 48 h, culture medium was replaced with 50 μL of the 3-(4,5-dimethylthiazol-2-yl)-2,5-diphenyl tetrazolium bromide (MTT) reagent (Sigma-Aldrich, St. Louis, MO, USA). Then, 2.5 h later, 100 μL of dimethyl sulfoxide (Scharlab Chemie, Barcelona, Spain) was added to each well, and the proliferation of cells was calculated by the absorbance at 570 nm with a microplate reader.

### 4.3. Western Blot Analysis

Cell lysis buffer containing protease (NaVO_3_, NaS, and Na_4_P2O_7_) (Sigma-Aldrich, St Louis, MO, USA) and phosphatase inhibitors (Leupton, Tosyllysine chloromethyl ketone (TLCK), Tosylsulphonyl phenylalanyl chloromethyl ketone (TPCK), Aport, and Phenylmethanesulfonyl fluoride (PMSF)) (Sigma-Aldrich, St Louis, MO, USA) was used for lysing cell samples. Cellular proteins (20 μg) were separated by sodium dodecylsulfate (SDS)-polyacrylamide gel electrophoresis (PAGE) and transferred to polyvinylidene difluoride (PVDF) membranes. Membranes were first probed by primary antibodies at 4 °C overnight, and the dilution of the primary antibodies was 1:1000. Secondary antibodies (at a 1:5000 dilution) were interacted with the membranes at room temperature for 1 h, and the bands were visualized with an enhanced chemiluminescence (ECL) detection reagent (Millipore, Billerica, MA, USA). Each experiment was conducted twice for verification. The following antibodies were used in the experiments: phosphorylated (p)- and total (T)-ERK (1:1000, Cell Signal #9101, #4695), P38 (1:1000, Cell Signal #4511, #8690), mitogen-activated protein kinase kinase (MEK) (1:1000, Cell Signal #2338, #9122), c-Jun N-terminal kinase (JNK) (1:1000, Cell Signal #4668, #9252), AKT (1:1000, Cell Signal #4060, #4685), zonula occludens (ZO)-1 (1:1000, Cell Signal #8193), N-cadherin (1:1000, Cell Signal #13116), c-Myc (1:1000, Cell Signal #5605), Snail (1:1000, Cell Signal #3879), PCNA (1:1000, Cell Signaling, Danvers, MA, USA #13110) and anti-α-tubulin (1:5000, Sigma-Aldrich #T9026). Immunoblot signals were quantified by densitometric scanning (ImageJ software 1.47v, National Institutes of Health, Bethesda, Rockville, MD, USA).

### 4.4. Wound-Healing Migration Assay

HMGCS2-modified Huh-7 (4.3 × 10^5^ cells/well) and HepG2 cells (10^6^ cells/well) were separately seeded in ibidi culture insert dishes (ibidi, Martinsried, Germany), and incubated for 24 h at 37 °C in a 5% CO_2_ incubator. Culture inserts were carefully removed with sterile tweezers and then filled with fresh culture medium together with various doses of sorafenib. The medium was renewed every 2 days. The cell sample was recorded every 24 h, and the experiment was terminated when the scratch in the middle with any dose of sorafenib was fully healed.

### 4.5. RNA Extraction and Quantitative Real-Time Polymerase Chain Reaction (QPCR)

TRIzol reagent (Ambion, Carlsbad, CA, USA) was used to extract total RNA. Complementary DNA (cDNA) was synthesized from 2 μg cellular RNA via High-Capacity cDNA Reverse Transcription Kits (Applied Biosystems, Carlsbad, CA, USA). 4 μL of cDNA along with 6 μL cocktail which containing 5μL KAPA SYBR^®^ FAST qPCR Master Mix, 0.5 μL specific forward primer, and 0.5 μL specific reverse primer. The 10 μL mixed sample was loaded into 48-well PCR plates, and was analyzed by StepOnePlus system (Applied Biosystems, Foster City, CA, USA). The data was exported into Excel for further measurement later on. The comparative cycle threshold (Ct) method with normalization to GAPDH was used to determine gene expression levels. The primers used for QPCR are listed as follows: GAPDH, forward, 5′-TCACCACCATGGAGAAGGC-3′. reverse, 5′-GCTAAGCAGTTGGTGGTGCA-3′. Cyclin D1, forward, 5′-AGGAACAGAAGTGCGAGGAGG-3′. reverse, 5′-GGATGGAGTTGTCGGTGTAGATG-3′. N-cadherin, forward, 5′-CGTGGAGGAGAAGAAGACCAG-3′. reverse, 5′-GCATCAGGCTCCACAGT-3′. Snail, forward, 5′-GCTGCAGGACTCAATCCAGA-3′. reverse, 5′-ATCTCCGGAGGTGGGATG-3′.

### 4.6. Statistical Analyses

All data are presented as the mean ± standard deviation (SD). Statistical calculations were performed using the SPSS v20.0 program (SPSS, Chicago, IL, USA). Differences between individual groups were determined by Student’s *t*-test, and *p* < 0.05 was considered statistically significant.

## 5. Conclusions

HCC is the most common primary malignant tumor worldwide. Sorafenib is a first-line drug for patients with advanced HCC. However, long-term treatment with sorafenib often results in reduced sensitivity of tumor cells to the drug, leading to acquired resistance. Our previous study demonstrated that decreased HMGCS2 expression was correlated with the severity of HCC; however, whether HMGCS2 loss mediation of KB reduction alters the sensitivity of human HCC to sorafenib treatment remains unclear. As shown in Figure 6, this study showed that HMGCS2 downregulation enhanced the proliferative ability and attenuated the cytotoxicity effects of sorafenib by activating expressions of p-ERK, p-P38, and p-AKT. In contrast, the cell proliferation was lower in HMGCS2 overexpressed cells and the sorafenib-induced cytotoxic effects was enhanced in HCC cells by inhibiting ERK activation. Furthermore, we showed that knockdown HMGCS2 promoted the migratory ability, inhibited the epithelial marker, ZO-1, and increased c-Myc expressions in both sorafenib-treated Huh7 and HepG2 cells. Although HMGCS2 overexpression did not alter the migratory effect, expressions of the mesenchymal markers, N-cadherin and c-Myc, decreased in sorafenib-treated HMGCS2-overexpressing cells. Finally, we investigated whether HMGCS2 expression influenced sorafenib sensitivity under the ketone treatment. We showed that β-HB pretreatment decreased the proliferative ability and enhanced the cytotoxic effects of sorafenib in HCC cells. Taken together, this study characterized the impacts of HMGCS2 expression and KB treatment on sorafenib usefulness in liver cancer cells.

## Data Availability

Requests for resources and reagents should be contacted to the corresponding author.

## Supplementary Materials

The following supporting information can be downloaded at: https://www.mdpi.com/article/10.3390/molecules27228015/s1, Figure S1: Low doses of β-HB pretreatment expressed no significant difference in cell proliferative and sorafenib sensitivity ability in Huh7, HepG2 and Hep3B cells.

## Sample Availability

Samples of the compounds are not available from the authors.

## References

[1] Singh S.K., Singh R. (2020). Liver cancer incidence and mortality: Disparities based on age, ethnicity, health and nutrition, molecular factors, and geography. Cancer Health Disparities.

[2] Siegel R.L., Miller K.D., Fuchs H.E., Jemal A. (2021). Cancer Statistics, 2021. CA Cancer J. Clin..

[3] Cha C., Dematteo R.P. (2005). Molecular mechanisms in hepatocellular carcinoma development. Best Pract. Res. Clin. Gastroenterol..

[4] Yang J.D., Hainaut P., Gores G.J., Amadou A., Plymoth A., Roberts L.R. (2019). A global view of hepatocellular carcinoma: Trends, risk, prevention and management. Nat. Rev. Gastroenterol. Hepatol..

[5] Zhu Y.J., Zheng B., Wang H.Y., Chen L. (2017). New knowledge of the mechanisms of sorafenib resistance in liver cancer. Acta Pharmacol. Sin..

[6] Kudo M., Finn R.S., Qin S., Han K.H., Ikeda K., Piscaglia F., Baron A., Park J.W., Han G., Jassem J. (2018). Lenvatinib versus sorafenib in first-line treatment of patients with unresectable hepatocellular carcinoma: A randomised phase 3 non-inferiority trial. Lancet.

[7] Shafqat N., Turnbull A., Zschocke J., Oppermann U., Yue W.W. (2010). Crystal structures of human HMG-CoA synthase isoforms provide insights into inherited ketogenesis disorders and inhibitor design. J. Mol. Biol..

[8] Porter W.H., Yao H.H., Karounos D.G. (1997). Laboratory and clinical evaluation of assays for beta-hydroxybutyrate. Am. J. Clin. Pathol..

[9] Zhang S., Xie C. (2017). The role of OXCT1 in the pathogenesis of cancer as a rate-limiting enzyme of ketone body metabolism. Life Sci..

[10] Grabacka M., Pierzchalska M., Dean M., Reiss K. (2016). Regulation of Ketone Body Metabolism and the Role of PPARalpha. Int. J. Mol. Sci..

[11] Puchalska P., Crawford P.A. (2017). Multi-dimensional Roles of Ketone Bodies in Fuel Metabolism, Signaling, and Therapeutics. Cell Metab..

[12] Vallejo F.A., Shah S.S., de Cordoba N., Walters W.M., Prince J., Khatib Z., Komotar R.J., Vanni S., Graham R.M. (2020). The contribution of ketone bodies to glycolytic inhibition for the treatment of adult and pediatric glioblastoma. J. Neurooncol..

[13] Yellen G. (2008). Ketone bodies, glycolysis, and KATP channels in the mechanism of the ketogenic diet. Epilepsia.

[14] Hashim S.A., VanItallie T.B. (2014). Ketone body therapy: From the ketogenic diet to the oral administration of ketone ester. J. Lipid Res..

[15] Jensen N.J., Wodschow H.Z., Nilsson M., Rungby J. (2020). Effects of Ketone Bodies on Brain Metabolism and Function in Neurodegenerative Diseases. Int. J. Mol. Sci..

[16] Tomita I., Kume S., Sugahara S., Osawa N., Yamahara K., Yasuda-Yamahara M., Takeda N., Chin-Kanasaki M., Kaneko T., Mayoux E. (2020). SGLT2 Inhibition Mediates Protection from Diabetic Kidney Disease by Promoting Ketone Body-Induced mTORC1 Inhibition. Cell Metab..

[17] Song J.P., Chen L., Chen X., Ren J., Zhang N.N., Tirasawasdichai T., Hu Z.L., Hua W., Hu Y.R., Tang H.R. (2020). Elevated plasma beta-hydroxybutyrate predicts adverse outcomes and disease progression in patients with arrhythmogenic cardiomyopathy. Sci. Transl. Med..

[18] Yurista S.R., Nguyen C.T., Rosenzweig A., de Boer R.A., Westenbrink B.D. (2021). Ketone bodies for the failing heart: Fuels that can fix the engine?. Trends Endocrinol. Metab..

[19] Otto C., Kaemmerer U., Illert B., Muehling B., Pfetzer N., Wittig R., Voelker H.U., Thiede A., Coy J.F. (2008). Growth of human gastric cancer cells in nude mice is delayed by a ketogenic diet supplemented with omega-3 fatty acids and medium-chain triglycerides. BMC Cancer.

[20] Allen B.G., Bhatia S.K., Buatti J.M., Brandt K.E., Lindholm K.E., Button A.M., Szweda L.I., Smith B.J., Spitz D.R., Fath M.A. (2013). Ketogenic diets enhance oxidative stress and radio-chemo-therapy responses in lung cancer xenografts. Clin. Cancer Res..

[21] Shukla S.K., Gebregiworgis T., Purohit V., Chaika N.V., Gunda V., Radhakrishnan P., Mehla K., Pipinos I.I., Powers R., Yu F. (2014). Metabolic reprogramming induced by ketone bodies diminishes pancreatic cancer cachexia. Cancer Metab..

[22] Wang Y.H., Liu C.L., Chiu W.C., Twu Y.C., Liao Y.J. (2019). HMGCS2 Mediates Ketone Production and Regulates the Proliferation and Metastasis of Hepatocellular Carcinoma. Cancers.

[23] Wang Y.H., Suk F.M., Liao Y.J. (2020). Loss of HMGCS2 Enhances Lipogenesis and Attenuates the Protective Effect of the Ketogenic Diet in Liver Cancer. Cancers.

[24] Ha T.Y., Hwang S., Moon K.M., Won Y.J., Song G.W., Kim N., Tak E., Ryoo B.Y., Hong H.N. (2015). Sorafenib inhibits migration and invasion of hepatocellular carcinoma cells through suppression of matrix metalloproteinase expression. Anticancer Res..

[25] Siddharth S., Kuppusamy P., Wu Q., Nagalingam A., Saxena N.K., Sharma D. (2022). Metformin Enhances the Anti-Cancer Efficacy of Sorafenib via Suppressing MAPK/ERK/Stat3 Axis in Hepatocellular Carcinoma. Int. J. Mol. Sci..

[26] Liang C.C., Park A.Y., Guan J.L. (2007). In vitro scratch assay: A convenient and inexpensive method for analysis of cell migration in vitro. Nat. Protoc..

[27] Torii S., Yamamoto T., Tsuchiya Y., Nishida E. (2006). ERK MAP kinase in G cell cycle progression and cancer. Cancer Sci..

[28] Chai H., Luo A.Z., Weerasinghe P., Brown R.E. (2010). Sorafenib downregulates ERK/Akt and STAT3 survival pathways and induces apoptosis in a human neuroblastoma cell line. Int. J. Clin. Exp. Pathol..

[29] Moon H., Ro S.W. (2021). MAPK/ERK Signaling Pathway in Hepatocellular Carcinoma. Cancers.

[30] Wang L., Guo W., Guan H., Yan N., Cai X., Zhu L. (2021). Local anesthetic bupivacaine inhibits proliferation and metastasis of hepatocellular carcinoma cells via suppressing PI3K/Akt and MAPK signaling. J. Biochem. Mol. Toxicol..

[31] Nagai T., Arao T., Furuta K., Sakai K., Kudo K., Kaneda H., Tamura D., Aomatsu K., Kimura H., Fujita Y. (2011). Sorafenib inhibits the hepatocyte growth factor-mediated epithelial mesenchymal transition in hepatocellular carcinoma. Mol. Cancer Ther..

[32] Xia H., Chen J., Shi M., Gao H., Sekar K., Seshachalam V.P., Ooi L.L.P., Hui K.M. (2015). EDIL3 is a novel regulator of epithelial-mesenchymal transition controlling early recurrence of hepatocellular carcinoma. J. Hepatol..

[33] Giannelli G., Koudelkova P., Dituri F., Mikulits W. (2016). Role of epithelial to mesenchymal transition in hepatocellular carcinoma. J. Hepatol..

[34] Rodriguez-Hernandez M.A., Chapresto-Garzon R., Cadenas M., Navarro-Villaran E., Negrete M., Gomez-Bravo M.A., Victor V.M., Padillo F.J., Muntane J. (2020). Differential effectiveness of tyrosine kinase inhibitors in 2D/3D culture according to cell differentiation, p53 status and mitochondrial respiration in liver cancer cells. Cell Death Dis..

[35] Louisa M., Suyatna F.D., Wanandi S.I., Asih P.B.S., Syafruddin D. (2016). Differential expression of several drug transporter genes in HepG2 and Huh-7 cell lines. Adv. Biomed. Res..

[36] Papachristou F., Anninou N., Koukoulis G., Paraskakis S., Sertaridou E., Tsalikidis C., Pitiakoudis M., Simopoulos C., Tsaroucha A. (2021). Differential effects of cisplatin combined with the flavonoid apigenin on HepG2, Hep3B, and Huh7 liver cancer cell lines. Mutat. Res. Genet. Toxicol. Environ. Mutagen..

[37] Yao A., Li Z., Lyu J., Yu L., Wei S., Xue L., Wang H., Chen G.Q. (2021). On the nutritional and therapeutic effects of ketone body D-beta-hydroxybutyrate. Appl. Microbiol. Biotechnol..

[38] Mikami D., Kobayashi M., Uwada J., Yazawa T., Kamiyama K., Nishimori K., Nishikawa Y., Nishikawa S., Yokoi S., Taniguchi T. (2020). beta-Hydroxybutyrate enhances the cytotoxic effect of cisplatin via the inhibition of HDAC/survivin axis in human hepatocellular carcinoma cells. J. Pharmacol. Sci..

[39] Shippy D.C., Wilhelm C., Viharkumar P.A., Raife T.J., Ulland T.K. (2020). beta-Hydroxybutyrate inhibits inflammasome activation to attenuate Alzheimer’s disease pathology. J. Neuroinflamm..

[40] Mascaro C., Buesa C., Ortiz J.A., Haro D., Hegardt F.G. (1995). Molecular cloning and tissue expression of human mitochondrial 3-hydroxy-3-methylglutaryl-CoA synthase. Arch. Biochem. Biophys..

[41] Wang J., Shidfar A., Ivancic D., Ranjan M., Liu L., Choi M.R., Parimi V., Gursel D.B., Sullivan M.E., Najor M.S. (2017). Overexpression of lipid metabolism genes and PBX1 in the contralateral breasts of women with estrogen receptor-negative breast cancer. Int. J. Cancer.

[42] Saraon P., Cretu D., Musrap N., Karagiannis G.S., Batruch I., Drabovich A.P., Van Der Kwast T., Mizokami A., Morrissey C., Jarvi K. (2013). Quantitative proteomics reveals that enzymes of the ketogenic pathway are associated with prostate cancer progression. Mol. Cell. Proteom..

[43] Camarero N., Mascaro C., Mayordomo C., Vilardell F., Haro D., Marrero P.F. (2006). Ketogenic HMGCS2 Is a c-Myc target gene expressed in differentiated cells of human colonic epithelium and down-regulated in colon cancer. Mol. Cancer Res..

[44] Lee Y.E., He H.L., Shiue Y.L., Lee S.W., Lin L.C., Wu T.F., Chang I.W., Lee H.H., Li C.F. (2015). The prognostic impact of lipid biosynthesis-associated markers, HSD17B2 and HMGCS2, in rectal cancer treated with neoadjuvant concurrent chemoradiotherapy. Tumour Biol..

[45] Su S.G., Yang M., Zhang M.F., Peng Q.Z., Li M.Y., Liu L.P., Bao S.Y. (2017). miR-107-mediated decrease of HMGCS2 indicates poor outcomes and promotes cell migration in hepatocellular carcinoma. Int. J. Biochem. Cell Biol..

[46] Dmitrieva-Posocco O., Wong A.C., Lundgren P., Golos A.M., Descamps H.C., Dohnalova L., Cramer Z., Tian Y., Yueh B., Eskiocak O. (2022). beta-Hydroxybutyrate suppresses colorectal cancer. Nature.

[47] Xiang Y., Wang M., Miao H. (2022). Ketogenic diet: New avenues to overcome colorectal cancer. Signal Transduct. Target. Ther..

[48] Winter S.F., Loebel F., Dietrich J. (2017). Role of ketogenic metabolic therapy in malignant glioma: A systematic review. Crit. Rev. Oncol. Hematol..

[49] Grabacka M.M., Wilk A., Antonczyk A., Banks P., Walczyk-Tytko E., Dean M., Pierzchalska M., Reiss K. (2016). Fenofibrate Induces Ketone Body Production in Melanoma and Glioblastoma Cells. Front. Endocrinol..

[50] Feng S., Wang H., Wang Y., Sun R., Xie Y., Zhou Z., Wang H., Aa J., Zhou F., Wang G. (2019). Apatinib induces 3-hydroxybutyric acid production in the liver of mice by peroxisome proliferator-activated receptor alpha activation to aid its antitumor effect. Cancer Sci..

[51] Weis E.M., Puchalska P., Nelson A.B., Taylor J., Moll I., Hasan S.S., Dewenter M., Hagenmuller M., Fleming T., Poschet G. (2022). Ketone body oxidation increases cardiac endothelial cell proliferation. EMBO Mol. Med..

[52] Liao Y.J., Wang Y.H., Wu C.Y., Hsu F.Y., Chien C.Y., Lee Y.C. (2021). Ketogenic Diet Enhances the Cholesterol Accumulation in Liver and Augments the Severity of CCl4 and TAA-Induced Liver Fibrosis in Mice. Int. J. Mol. Sci..

[53] O’Neill B., Raggi P. (2020). The ketogenic diet: Pros and cons. Atherosclerosis.

[54] Stubbs B.J., Cox P.J., Evans R.D., Cyranka M., Clarke K., de Wet H. (2018). A Ketone Ester Drink Lowers Human Ghrelin and Appetite. Obesity.

[55] Wiers C.E., Vendruscolo L.F., van der Veen J.W., Manza P., Shokri-Kojori E., Kroll D.S., Feldman D.E., McPherson K.L., Biesecker C.L., Zhang R. (2021). Ketogenic diet reduces alcohol withdrawal symptoms in humans and alcohol intake in rodents. Sci. Adv..

[56] Poff A.M., Koutnik A.P., Egan B. (2020). Nutritional Ketosis with Ketogenic Diets or Exogenous Ketones: Features, Convergence, and Divergence. Curr. Sports Med. Rep..

[57] Poff A.M., Ari C., Arnold P., Seyfried T.N., D’Agostino D.P. (2014). Ketone supplementation decreases tumor cell viability and prolongs survival of mice with metastatic cancer. Int. J. Cancer.

[58] Chen J., Hou H., Chen H., Luo Y., He Y., Zhang L., Zhang Y., Liu H., Zhang F., Liu Y. (2019). Identification of beta-hydroxybutyrate as a potential biomarker for female papillary thyroid cancer. Bioanalysis.

[59] Huang C.K., Chang P.H., Kuo W.H., Chen C.L., Jeng Y.M., Chang K.J., Shew J.Y., Hu C.M., Lee W.H. (2017). Adipocytes promote malignant growth of breast tumours with monocarboxylate transporter 2 expression via beta-hydroxybutyrate. Nat. Commun..

[60] Rodrigues L.M., Uribe-Lewis S., Madhu B., Honess D.J., Stubbs M., Griffiths J.R. (2017). The action of beta-hydroxybutyrate on the growth, metabolism and global histone H3 acetylation of spontaneous mouse mammary tumours: Evidence of a beta-hydroxybutyrate paradox. Cancer Metab..

[61] Chen K.F., Chen H.L., Tai W.T., Feng W.C., Hsu C.H., Chen P.J., Cheng A.L. (2011). Activation of phosphatidylinositol 3-kinase/Akt signaling pathway mediates acquired resistance to sorafenib in hepatocellular carcinoma cells. J. Pharmacol. Exp. Ther..

[62] Su J.C., Tseng P.H., Wu S.H., Hsu C.Y., Tai W.T., Li Y.S., Chen I.T., Liu C.Y., Chen K.F., Shiau C.W. (2014). SC-2001 overcomes STAT3-mediated sorafenib resistance through RFX-1/SHP-1 activation in hepatocellular carcinoma. Neoplasia.

