# Supplementary material for: HMGCS2 Mediation of Ketone Levels Affects Sorafenib Treatment Efficacy in Liver Cancer Cells
Source: Molecules. 2022 Nov 18;27(22):8015. doi: 10.3390/molecules27228015 (PMC9697984; doi:10.3390/molecules27228015)
Supplement: Supplementary file 1 [file molecules-27-08015-s001.zip › molecules-1960827-supplementary.pdf]

HMGCS2 mediation of ketone levels affects sorafenib treatment efficacy in liver cancer cells

Fat-Moon Suk[1,2,#], Chien-Ying Wu[3,#], Wan-Chun Chiu[4,5,6], Chia-Ying Chien[3], Tzu-Lang Chen[7], Yi-Jen Liao[3,*]

Figure S1

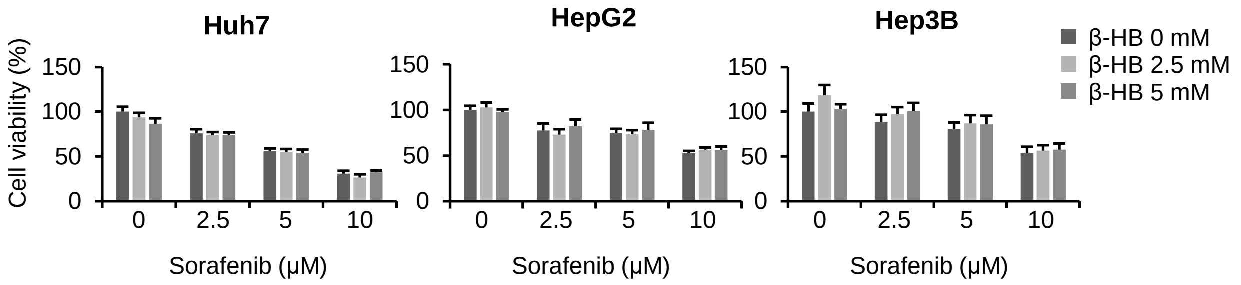

Huh7, Hep3B, and HepG2 cells were maintained in β-HB (0, 2.5, 5 mM)- supplemented medium for 2 days, and then cells were re-seeded in 96-well plates and treated with 0, 2.5, 5, and 10 μM sorafenib for 48 h. An MTT assay was conducted to detect cell viability.
